# The Role of Social Context in Physiological and Psychological Restoration in a Forest: Case Study of a Guided Forest Therapy Program in Taiwan

**DOI:** 10.3390/ijerph181910076

**Published:** 2021-09-25

**Authors:** Chia-Pin Yu, Heng-Ting Chen, Pei-Hua Chao, Jie Yin, Ming-Jer Tsai

**Affiliations:** 1 School of Forest and Resource Conservation, National Taiwan University, Taipei 10617, Taiwan; simonyu@ntu.edu.tw (C.-P.Y.); r05625010@ntu.edu.tw (H.-T.C.); tmj@ntu.edu.tw (M.-J.T.); 2 The Experimental Forest, College of Bioresources and Agriculture, National Taiwan University, Nantou 55704, Taiwan; 3 Department of Environmental Health, Harvard T.H. Chan School of Public Health, Boston, MA 02138, USA; jieyin@hsph.harvard.edu; 4 Department of Bio-Industry Communication and Development, National Taiwan University, Taipei 10617, Taiwan; 5 College of Architecture and Urban Planning, Tongji University, Shanghai 200092, China

**Keywords:** forest therapy, restorative benefits, physiological and psychological responses, guided program, field experimental design

## Abstract

Existing studies have demonstrated the restorative benefits of being in forests. However, most studies have designed participants to engage individually in forest walking and viewing, which neglects the social aspect of conversation. Researchers suggested that social context should be studied in order to have a better understanding how forests foster human health. To this end, we examined the role of social context using three types of forest therapy programs: a guided program, a self-guided program, and a walk alone program. A between-subject, pretest–posttest field experimental design was employed to evaluate restorative effects by measuring the physiological responses and mood states incurred in different forest therapy programs. Our findings showed, that the walk alone group exhibited a significant systolic blood pressure decrease and a significant increase in sympathetic nervous activity; the self-guided group showed a significant increase in heart rate values and significant decreases in systolic blood pressure and diastolic blood pressure; and the guided group revealed a significant decrease in systolic blood pressure. Further, the three forest therapy programs had positive effects on improving mood states, except a nonsignificant vigor–activity increase in the walk alone group. The three programs did not exhibit significant differences in changes of restorative benefits in physiological and psychological measures except for a significant difference in changes in sympathetic nervous activity between the walk alone group and guided group. The results showed the restorative benefits of forest therapy are apparent regardless of the program type. The management team should continue promoting forest therapy for public health by providing different types of forest therapy programs and experiences.

## 1. Introduction

Convergent evidence indicates connecting with natural environments, such as forests, has proven to be an effective approach to reduce mental health symptoms and physiological stress [1,2,3,4,5,6,7,8]. The amount of scientific evidence highlighting the health benefits of interacting with nature is continually increasing. Miyazaki, Song, and Ikei [9] discussed nature therapy as an intervention to prevent negative health outcomes via increasing physiological relaxation and strengthening the immune system. More specifically, immersion in natural environments helps to reduce stress levels and improve immune response, suggesting nature therapy can improve an individual’s resistance to disease. Among all types of nature therapy, forest therapy has been discussed at length and there is substantial scientific evidence supporting its positive effects on human health [9]. Being in a forest can reduce an individual’s stress levels and improve their physical and mental health. The Ministry of Agriculture, Forestry and Fisheries in Japan coined a word, shinrin-yoku (translates literally as “forest bathing”), describing it as a process people use to restore balance and health by immersing themselves in a forest environment and absorbing its atmosphere [10]. The term “forest therapy,” derived from shinrin-yoku, describes the medically proven health effects resulting from exposure to forests [11] (p. 2533). Additionally, Korean researchers defined forest therapy as “immune-strengthening and health-promoting activities utilizing various elements of the forest, such as fragrance and scenic view” [12] (p. 274). In Taiwan, we consider forest therapy to be a therapeutic recreational activity using forest resources to improve human health and well-being, as well as to promote a sustainable lifestyle that contributes to the human–nature balance [13]. Because of the ample evidence supporting its health effects, forest therapy has gained popularity and has become recognized globally as an effective method for reducing stress levels and promoting population health [14,15].

Forest therapy research applies an evidence-based approach and field experiments to evaluate the health outcomes of participants. Empirical studies have demonstrated the beneficial health effects on mental health of being in forests [2,6,16,17,18,19,20,21]. Those studies indicated that in forest environments the intensity of participants’ negative emotions decreased while their positive emotions increased, as compared to the emotions of people in urban settings. For instance, Park et al. [20] implemented a large-scale field study in Japan, recruiting 168 participants from 14 forests and 14 urban areas and administering the Profile of Mood States (POMS) questionnaire to evaluate the psychological effects of forest therapy. They learned participants’ levels of vigor were significantly higher and their negative emotions were lower in the forest, as compared to people in the urban settings. Similar results were reported by Lee et al. [19] and Takayama et al. [21], who both reported that immersion in forests increased feelings of vigor, subjective recovery, and vitality and decreased levels of anxiety, as compared with immersion in urban settings. In addition, studies have revealed positive relationships between forest therapy and physical health. For instance, forest therapy reduces pulse rate and blood pressure [3,5,6,8,11,16,22,23,24,25,26], increases parasympathetic nervous system (PSNS) activity, reduces sympathetic nervous system (SNS) activity [10,18,19,22,23,27], and improves immune system response [28,29,30]. The findings have proven psychological and physiological restoration of being in a forest and these indicators turn out a standard in forest therapy research.

Previous studies have illustrated the mental and physical health effects of forest therapy. However, most studies have asked participants to engage individually in forest walking and viewing in order to limit the effects of confounding factors (e.g., company or social interaction), which neglects the social aspect of conversation in forest therapy. In other words, rarely has recent forest therapy literature discussed social context or explored the potential effects of interaction between participants or between participants and a guide on psychological and physiological outcomes. Table 1 lists research with two experiential types and the major studies using solo activities in their experimental design. In actual forest therapy practice, a guide typically leads the way in order to ensure the safety of participants and helps to immerse them in the forest through planned activities. Guided forest therapy research (see Table 1) has demonstrated forest therapy programs can have substantial physiological and psychological benefits for selected groups. For example, in a one-group pretest–posttest field experimental design, a guided forest therapy program resulted in a significant decrease in pulse rate and blood pressure, alleviated negative emotions, and improved positive affect [31]. Although those results are promising, because of the lack of a walk alone comparison in the study, it remains unclear whether a guide can contribute to the benefits of forest therapy programs.

Meyer and Bürger-Arndt [35] suggested that social context should be studied so as to better understand how forests foster human health. To our knowledge, a limited amount of research has been performed quantifying the health effects of human company (e.g., [36,37]). Staats and Hartig [37] concluded that company creates a feeling of safety that can facilitate restoration; however, after controlling for safety, they found that walking alone also can enhance restoration. In other words, they explained two opposite effects of company in a natural environment, that (a) company enables restoration through a pathway for safety, and (b) solitude enhances restoration when safety is satisfied. Igawahara et al. [36] investigated the physiological and psychological responses of participants in a guided forest bathing program and compared those with a walk alone program. Their results suggested that walking with a guide produces stronger therapeutic and relaxation effects than does walking alone. Both of these studies illustrate the relationship between company and a sense of safety, so it can be said from a social context aspect that those two factors in combination enhance the restorative quality of a nature experience. Urban dwellers may experience feelings of insecurity and discomfort when visiting forests because of their unfamiliarity, but a guide could mitigate these feelings, possibly contributing to stress relief and a mood state change [36] (p. 600). Therefore, a guided forest therapy program may promote restoration by nurturing a feeling of safety [36,37,38]. Moreover, guides play a role in providing enjoyable and immersive experiences in forests through interpretation and leading of therapeutic activities. These social interaction and fun activities yield a positive affective experience that contributes to restorative benefits [39]. Conversely, the direct attention of participants may be occupied as focus is required to follow a guide’s instructions, and that may degrade the quality of the restorative experience as compared to walking alone [38,40]. Anecdotally, a participant having followed a guided forest therapy activity in a previous study provided this feedback: “the guide was professional and excellent, however sometimes I felt he was interrupting my connection with the forest.” This intriguing feedback inspired the following question: “when promoting forest therapy, should we encourage people to experience forests alone or with a guide?” In other words, “does a guided program enhance the restorative benefits of forest therapy?” is an interesting and practical question in the study of forest therapy that remains unanswered because of a lack of evidence supporting or contradicting it. 

To address this question, in this exploratory study we examined the role of social context using three types of forest therapy programs: a guided program, a self-guided program, and a walk alone program supported by the Experimental Forest of National Taiwan University. We then investigated the restorative benefits using the selected forest therapy programs as a between-subject, pretest–posttest field experimental design.

## 2. Materials and Methods

### 2.1. Study Site

This study was conducted in the Xitou Nature Education Area (XNEA), Taiwan which is managed by the Experimental Forest of National Taiwan University. The Experimental Forest was created in 1901 with four primary objectives, namely academic research, environment education, natural resource conservation, and forest management demonstration. XNEA is located in a concave valley surrounded on three sides by mountains, covers approximately 2200 hectares, and ranges from 800 to 2000 m in elevation. The planted forest mainly consists of *Cryptomeria japonica* and *Phyllostachys pubescens*, with stand ages ranging from 40 to 90 years. The local temperature ranges from 11.0 to 20.8 °C (with an annual average temperature of 16.6 °C) and the relative humidity ranges from 88% to 93%. XNEA is a popular forest recreation destination in Taiwan and receives approximately two million visitors per year [41]. In addition, a well-established forest trail system has enabled XNEA to become an ideal location for forest therapy [42]. Today, the Experimental Forest plays a crucial role in promoting forest therapy in Taiwan, and forest therapy has become a primary focus of XNEA in its attempts to improve public health. Visitors can experience forest bathing alone or they can reserve a spot in a guided forest therapy program. The guided programs aim to improve the mental and physical health of participants through several therapeutic recreation activities, which entail stimulation of four senses: vision (observing different landscapes), hearing (listening to the sounds of birdsongs and running streams), olfaction (smelling wood and air), and touch (tangibly feeling cypress and the surfaces of leaves and trees). However, due to the limited capacity of the guided forest therapy programs, self-guided programs using designated maps to promote the sensory experiences are offered as an alternative for unguided visitors.

### 2.2. Experimental Design

A between-subject, pretest–posttest field experimental design was employed to evaluate the physiological responses and mood states incurred in different forest therapy programs. All participants were randomly assigned into the three study programs. Only the investigators know which program the participant is receiving. The single-blind design makes the results of study less likely to be biased. Three types of two hours forest therapy programs were designed for this study: a walk alone program (Control (C) group), a self-guided map program (Map (M) group), and a guided program (Guided (G) group). The C group was asked to freely explore the forest and to leisurely walk alone. The M group received a portable map to guide themselves alone, while the G group was led by a tour guide. The map illustrated the route and provided instructions of the activities that participants in the M group were solicited to follow. The participants of the M group were asked to engage in multisensory (visual, auditory, olfactory, and tactile) activities in the selected locations by following the instructions on the self-guided map; the following activities were included to encourage immersion in nature: observing the forest landscapes, listening to birdsongs and the river, inhaling and exhaling forest air, and embracing trees. The route and activities for the M and G groups were identical and the primary difference between these two groups was the presence of a guide. 

**Figure 1:**
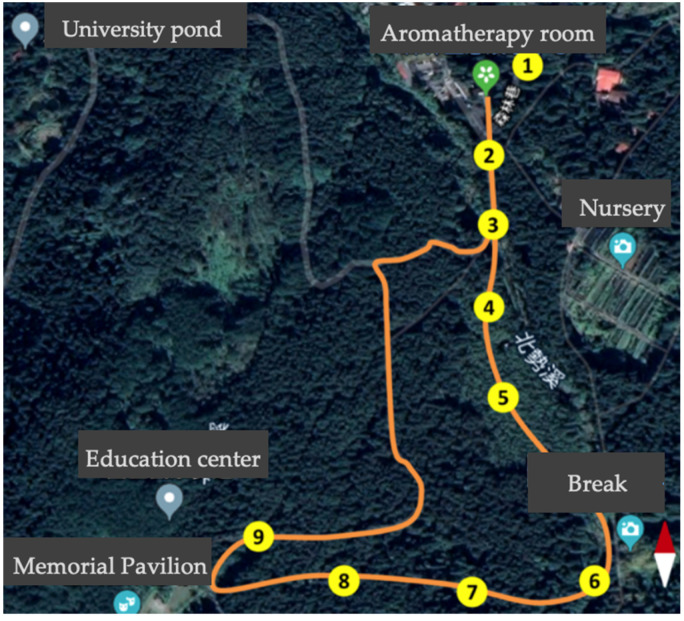
Map of forest therapy activities with locations.

The guided forest therapy program involved a three-kilometer journey at 1100–1200 m above sea level with an average walking speed of 2 km per hour. The program included multisensory experience activities, namely visual (observing the forest landscapes and looking for various colors), auditory (listening to the birdsongs and river), olfactory (inhaling and exhaling forest air), and tactile (embracing trees and touching bark) activities that helped to immerse the participants and to connect them with nature. Each activity was conducted at the same location to ensure participants in each group had a similar experience. To avoid stylistic differences between guides, all the guided programs were led by the same guide. The guided program’s time frame and locations are presented in Table 2 and Figure 1. Further, we controlled participant number at approximately six people to have a quality experience and to avoid a long waiting time of evaluation in the guided group.

**Figure 2:**
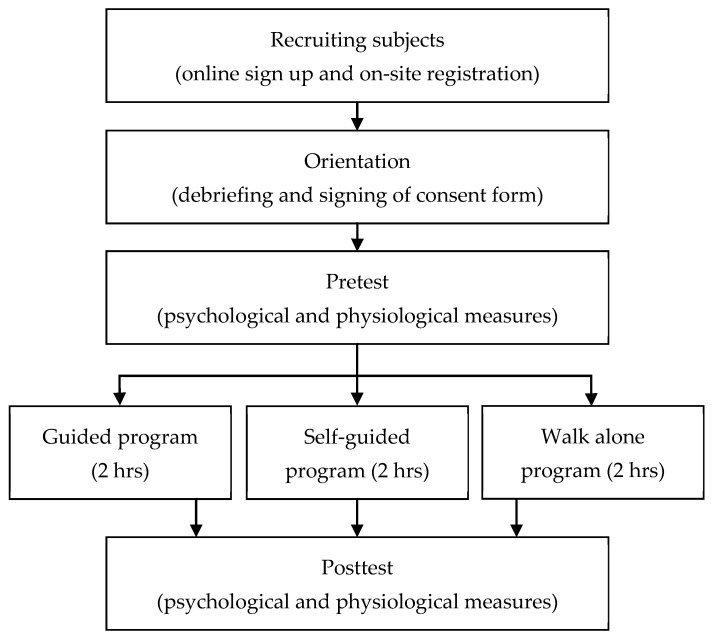
Experimental procedure.

The experiment began with an orientation session in an indoor facility, during which researchers briefly explained the experiment and then asked the participants to sign a consent form to reflect that the participants understood and agreed to participate in the study. In the pretest stage, the participants filled out a pretest questionnaire, which included demographic information and questions regarding emotional status. Then, participants’ physiological measurements were recorded, after which participants were randomly assigned to one of the three programs: (1) the C group freely strolled through the forest and returned to the facility by an appointed time; (2) the M group was asked to follow the designated route displayed in the map, visit marked locations on the forest therapy map for specific activities, and return to the facility by an appointed time; and (3) the G group was led through the forest therapy program by an instructor who guided participants in experiencing nature. After finishing the program, all participants filled out the posttest questionnaire and again had their physiological measurements recorded (Figure 2). The programs for all three groups were restricted to approximately two hours.

### 2.3. Participants

The experiment was promoted on-site at XNEA and through an online platform. Participants could either sign up online or register at XNEA to participate in the forest therapy experiment. The participants were required to be at least 20 years old and physically capable of completing the selected program. Consumption of tea, coffee, or other caffeinated drinks was not allowed during the day of the experiment until after the program was completed. In total, 99 participants were recruited and randomly assigned to the three two-hour programs. The study period was from 26 July–12 August 2018. Due to rain-related cancellations, the experiment was conducted over thirteen days. There were two sessions per day, one of which was a morning session from 8:00 a.m. to 12:30 p.m. and the other an afternoon session from 13:30 p.m. to 17:00 p.m. The study was approved by the Research Ethics Office of National Taiwan University (NTU-REC No. 201607HS008).

### 2.4. Physiological Measures

Heart rate (HR), systolic blood pressure (SBP), diastolic blood pressure (DBP), sympathetic nervous system (SNS) activity, parasympathetic nervous system (PSNS) activity, and sympathovagal balance (balance between the SNS and PSNS) were measured using the Quantitative Heart Rate Variability (QHRV; Medeia, Bulgaria) tool, which is validated and approved by the U.S. Food and Drug Administration. The QHRV is portable and easy to use and can evaluate and record these physiological measures in five minutes. These physiological measures are considered to be important indicators of human health and are in wide use in forest therapy studies (e.g., 7, 11, 12, 18). The autonomic nervous system consists of the SNS and PSNS activities. A higher level of SNS activity reflects that an individual is in a stressed state. Conversely, when a stressor is removed PSNS activity is triggered in order to return to an unstressed state, and PSNS is involved in regulating body function while in a resting state [43]. In forest therapy studies, the activities of SNS and PSNS have been evaluated using power spectral analysis of heart rate variability (HRV) and quantification of low frequency (LF, 0.04–0.15 Hz, representing SNS) and high frequency (HF, 0.15–0.40 Hz, representing PSNS) power. Nevertheless, concerning respiratory activity influences evaluation of SNS and PSNS activities and suggests more accurate measures by incorporating respiratory activity analysis with concurrent HRV analysis, yielded new indicators, namely LFa and RFa, that provide more accurate results of the functioning of SNS and PSNS, respectively [44,45,46,47]. Specifically, RFa is the average MIT-standard respiratory frequency area (beats per minute^2^/Hz), which is a measure of PSNS tone, and represents the frequency ranges associated with respiratory sinus arrhythmia (the cardiovagal response) and measures PSNS activity from higher frequency areas of the HRV spectrum. LFa is the average MIT-standard low frequency area (beats per minute^2^/Hz), which is a measure of SNS tone as mediated by PSNS tone. LFa/RFa ratio represents sympathovagal balance (SB) and is the average MIT-standard ratio (unitless), which is a measure of ANS balance [44,45,46,48,49]. The normal ranges of RFa and LFa for adults are from 1.0 to 10.0 bpm^2^, respectively. The normal range for SB (LFa/RFa) is 0.4 < SB < 3.0 [46], (p. 125). A higher or lower score may associate with a health issue. For measuring autonomic nervous system (ANS) activity in the current study, both indicators, LFa and RFa, reported by QHRV, were used for representing sympathetic nervous activity and parasympathetic nervous activity, respectively, and sympathovagal balance (LFa/RFa) was calculated accordingly.

### 2.5. Psychological Measures

We employed the short form of the POMS (POMS-SF) scale [50] with satisfactory validity and reliability, which has been widely used in forest therapy studies in order to examine the psychological restorative effects resulting from mood state changes [19,20,21]. This scale is composed of six constructs with 37 adjectives and used to measure emotional state in the dimensions of tension–anxiety (6 items), anger–hostility (7 items), fatigue–inertia (5 items), depression–dejection (8 items), confusion–bewilderment (5 items), and vigor–activity (6 items). The POMS-SF scale is scored on a 5-point Likert scale (0 = not at all; 4 = extremely). The sum of points received for items in one dimension is the total score for that dimension, so a high total score indicates a high level of the emotion represented by that dimension.

### 2.6. Statistical Analyses

The data were compiled, coded, and crosschecked in order to minimize human error. The statistical methods in this study include descriptive statistics, paired-sample t-test, and one-way ANOVA. The descriptive analysis results of major study variables were represented as mean ± standard deviation. Pretest–posttest comparisons of physiological and psychological measures across the three groups were completed using paired-sample t-tests. For comparing the restorative effects among the programs (G, M, and C groups), we used one-way ANOVAs on gain (or loss) scores [51] and the changes in percentages were reported. The significance level in this study was 0.05 and effect size was measured using partial eta squared (ηp^2^). All analyses were executed with SPSS 20.0 (IBM Corporation, NY, USA). Further, Levene’s homogeneity was checked for all pretest scores among three groups and revealed the variance of these scores were equal across groups.

## 3. Results

### 3.1. Demographic Information

The demographics of the 99 study participants are presented in Table 3. It is noted that 37, 34, and 28 participants were randomly assigned to the C, M, and G groups, respectively. Overall, 40 men (40.4%) and 59 women (59.6%) participated, with 38 people aged 21–30 years (38.4%), 14 aged 31–50 years (14.1%), 41 aged 51–65 years (41.4%), and 6 aged 66 years and older (6.1%). The average age was 43.7 years, with a standard deviation of 16.7 years. Nearly 76% of participants had a college degree. Most of the participants lived in either urban or suburban areas and had visited natural spaces within the past month. The pre-and-post physiological and psychological measurements of the three groups are displayed in Table 4. Because of device failure and time constraints, physiological data of 12 participants in the C group and one participant in the M group were incomplete, so those participants were excluded from the analysis of physiological responses. 

### 3.2. Influence of Programs on Physiological Responses

The results of paired-sample t-tests that were used to determine whether or not the changes in physiological responses were significant are presented in Table 5. The results of one-way ANOVAs on gain (or loss) scores for cross-group comparison and the amounts of change are reported in Table 6.

**Figure 3:**
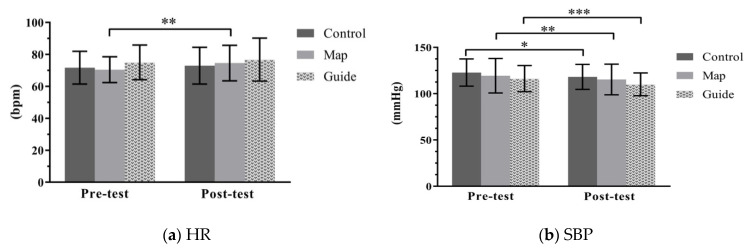

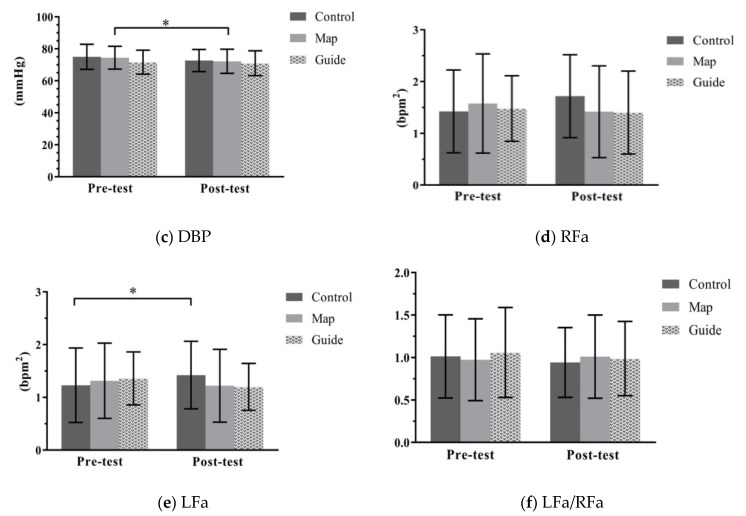
Pretest–posttest comparisons of physiological measures across the three groups. Abbreviations: (**a**) HR, heart rate; (**b**) SBP, systolic blood pressure; (**c**) DBP, diastolic blood pressure; (**d**) RFa, respiratory frequency area (parasympathetic nervous system activity measure); (**e**) LFa, low frequency area (sympathetic nervous system activity measure); (**f**) LFa/RFa (measure of sympathovagal balance). Note: Error bars depict standard deviation. *p < 0.05, ** *p* < 0.01, *** *p* < 0.001.

The pre-and-post measures of physiological responses are displayed in Figure 3. Regarding the heart rate (HR) of participants, the self-guided (M) group exhibited significantly higher posttest HRs than pretest HRs (*t* = 3.413, *p* = 0.002). The changes observed in each group were 1.73% in the walk (C) group, 5.12% in the self-guided (M) group, and 2.24% in the guided (G) group. The differences in HR change were not significant among these groups (F = 1.527, *p* = 0.223, η_p_^2^ = 0.035). The posttest systolic blood pressure (SBP) scores for all groups were significantly lower than the pretest SBP scores: t = −2.741 (*p* = 0.011), *t* = −2.832 (*p* = 0.008), and *t* = −4.360 (*p* < 0.001) in the C, M, and G groups, respectively; the changes were −3.78%, −3.04%, and −5.31% in the C, M, and G groups, respectively. The changes in SBP among the three groups were not significant (F = 0.594, *p* = 0.555, η_p_^2^ = 0.014). For diastolic blood pressure (DBP), the M group exhibited a significant decrease (*t* = −2.502, *p* = 0.018). The changes in DBP value were −3.09%, −2.70%, and −0.93% in the C, M, and G groups, respectively. The decreases were not statistically significant among the three groups (F = 0.852, *p* = 0.430, η_p_^2^ = 0.020). An increase in RFa value was observed in the C group, while decreases were exhibited in both the M and G groups; however, comparisons of pretest and posttest values in RFa were not significant in all of the groups. The changes in RFa were 21.13%, −10.76%, and −5.41% in the C, M, and G groups, respectively. There was no significant difference in the changes among the three groups (F = 3.102, *p* = 0.05, η_p_^2^ = 0.070). A significant increase was observed in the C group’s posttest LFa (*t* = 2.184, *p* = 0.039). The changes in LFa were 15.45%, −6.87%, and −11.76% in the C, M, and G groups, respectively. Significant differences of change in LFa were found among the three groups (F = 3.552, *p* = 0.033, η_p_^2^ = 0.079), and the post hoc results revealed that the change in LFa of the C group was significantly higher than the difference in the G group. No group exhibited any significant pretest–posttest difference in autonomic balance (LFa/RFa), and the changes between groups were not significantly different (F = 0.527, *p* = 0.592, η_p_^2^ = 0.013).

### 3.3. Influence of Programs on Psychological Response

The paired-sample t-test results of emotional states are presented in Table 7. The differences between pretest and posttest were significant in all psychological responses except for a nonsignificant vigor–activity increase in the C group. The differences in mood state change scores among the three programs using one-way ANOVA and the changes are listed in Table 8. There were no observed differences among the three programs.

**Figure 4:**
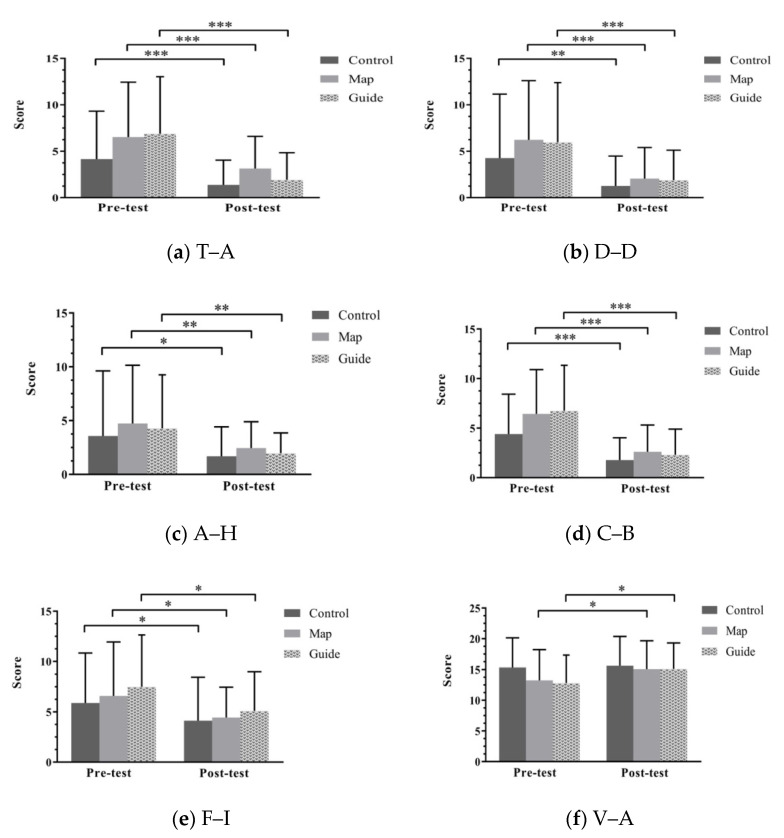
Pretest–posttest comparisons of psychological measures across the three groups. Abbreviations: (**a**) T–A, tension–anxiety; (**b**) D–D, depression–dejection; (**c**) A–H, anger–hostility; (**d**) C–B, confusion–bewilderment; (**e**) F–I, fatigue–inertia; (**f**) V–A, vigor–activity. Note: * *p* < 0.05, ** *p* < 0.01, *** *p* < 0.001.

The pre-and-post measures for each domain of POMS are displayed in Figure 4. In general, participants’ moods improved substantially and consistently across the different domains. Specifically, the mean tension–anxiety scores after the forest therapy decreased significantly by 66.83%, 51.76%, and 71.99% in the C, M, and G groups, respectively. Similarly, the mean depression–dejection scores decreased by 70.26%, 69.99%, and 68.29%, the mean anger–hostility scores decreased by 52.94%, 45.41%, and 54.31%, the mean confusion–bewilderment scores decreased by 59.64%, 59.32%, and 65.63%, and the mean fatigue–inertia scores decreased by 29.71%, 32.63%, and 31.87% in the C, M, and G groups, respectively. In addition, the mean vigor–activity score increases were significant in the M and G groups (with 13.8% and 18.1% increases in the M and G groups, respectively), but not significant in the C group (with 1.76% increase). The G group exhibited the largest improvement in tension–anxiety (−71.99%), anger–hostility (−54.31%), and confusion–bewilderment (−65.63%) for negative emotions and the largest enhancement in vigor–activity (18.10%) for positive emotions.

## 4. Discussion

### 4.1. Influences of the Three Forest Therapy Programs on Physiological Responses

Comparisons between pretest and posttest physiological measurements revealed differences in the restorative outcomes of the three study groups. Physiologically, the C group exhibited a significant SBP decrease and a significant increase in SNS activity, the M group exhibited a significant increase in HR values and significant decreases in SBP and DBP, and the G group exhibited a significant decrease in SBP. The significant SBP decrease in all three groups is consistent with the findings of previous studies (e.g., [31]) that indicate the benefit of these experiences to systolic blood pressure functions. Concerning the significant increase in SNS activity in the C group and the significant increase in HR in the M group, one possible explanation is that walking distance and speed influenced the results. Specifically, the forest therapy program combines immersion in a forest environment and walking. Immersion is expected to result in relaxation, which will suppress sympathetic nervous activity, activate parasympathetic nervous activity, and reduce HR. However, physical exercise (i.e., walking and hiking) required energy expenditure of the participants and may have boosted both sympathetic nervous activity and HR. The study did not control participants’ walking speeds in the M group; thus we speculate the increase of HR may be because they took a fast-paced walk during the experiment. In the C group, participants walked as they desired in the forest; so, if they chose to walk a long distance or to walk up a steep hill during the program, this would increase their sympathetic nervous activity.

### 4.2. Influences of the Three Forest Therapy Programs on Psychological Responses

Aside from a nonsignificant vigor–activity increase in the C group, all groups exhibited significant changes in each variable of the emotional aspect. In general, the three forest therapy programs had positive effects on improving mood states. The improvement of mood state changes across the three groups is consistent with the general view in prior studies that immersion in a forest contributes to improved mental health [7,11,16,17,18,19,20,21]. Concerning the nonsignificant vigor–activity increase in the C group, we found a relatively high pretest mean score in the C group (mean = 15.4) compared to the M and G groups (13.3 and 12.8 in the M and G groups, respectively). However, Levene’s homogeneity test revealed the variance for the pretest vigor–activity score was equal across groups (*p* = 0.468). 

### 4.3. Comparisons of Changes in Physiological Responses and Mood States among the Three Programs

The three programs in the study—walk alone (the C group), self-guided (the M group), and guided (the G group)—did not exhibit significant differences in changes of restorative benefits in physiological and psychological measures except for a significant difference in changes in sympathetic nervous activity (LFa) between the C group and G group. From a theoretical perspective, a guided forest therapy program could potentially promote restoration by nurturing a feeling of safety [36,37,38]. Staats and Hartig [37] indicated a feeling of safety influences restorative effects. In our study, we found no significant differences for most pre–post physiological changes (i.e., SBP, DBP, HR, RFa, and LFa/RFa) and changes of emotional responses across the three programs. Based on the results, we postulate that this may be because XNEA is a popular and well-organized forest recreation destination, which promotes feelings of security and comfort to all visitors. Therefore, the guided program simply may not be able to have a significant impact on feelings of security and safety, explaining the lack of significant differences in restorative effects. If that was the case, the variations in feelings of security and safety among the three programs would have been similar, and that may have resulted in the nonsignificant results for the between-group comparisons.

The changes in sympathetic nervous system (SNS) activity in the C group were significantly higher than the differences in the G group. For the C group, we speculate this increase in sympathetic nervous activity may be because participants engaged in more strenuous walking, either by walking a longer distance, walking at a faster speed, or hiking on a more highly graded route, each of which could potentially induce higher levels of sympathetic nervous system activity. The guided forest therapy program set the walking speed of participants at 2 km/h and provided an enjoyable and immersive experience in the forest via instructions for multisensory experiences that helped participants to relax, which reduced their level of sympathetic nervous activity. 

Although the three programs in the study did not exhibit significant differences in changes of restorative benefits in psychological measures, our analysis revealed that the guided group exhibited the most improvement in tension–anxiety (−71.99%), anger–hostility (−54.31%), and confusion–bewilderment (−65.63%), as well as the greatest increase in vigor–activity (18.10%). Therefore, it showed the guided forest therapy program performs better on mitigating negative emotions and improved positive moods.

We compared three forest therapy program types and found the restorative benefits of forest therapy in XNEA are apparent, regardless of the program type, particularly concerning mental health; the management team should continue promoting forest therapy for public health by providing different types of forest therapy programs and experiences.

### 4.4. Limitations of the Study and Future Research

Our study has a few limitations. First, we did not measure participants’ feelings of safety in the study. Since feelings of safety can potentially serve an important factor in explaining restorative effects, this variable should have been included to better understand its moderating effect on the restorative effects of the three programs. We suggest future forest therapy research add measures of feelings of safety or risk. Second, we did not control for the intensity of the exercise, which is a factor that may have influenced the physiological outcomes. Considering intensity of walk influences positive effects and happiness [52]; future studies should consider intensity of exercise (i.e., walking speed and degree of energy expenditure) in forest therapy program research. Moreover, the type of physical activity may affect the restorative effects, and that should be further explored; for example, forest yoga and Nordic walking are two activities that could be included in guided forest therapy programs, but the physiological and psychological responses resulting from the two activities may drastically differ. Third, environmental conditions such as illumination, humidity, and temperature, together with forest landscapes, potentially influence restorative effects. These and their associations are worth investigating in future studies.

## 5. Conclusions

Our study revealed that the walk alone group exhibited a significant SBP decrease and a significant increase in SNS activity, the self-guided group exhibited a significant increase in HR values and significant decreases in SBP and DBP, and the guided group showed a significant decrease in SBP. Further, the three forest therapy programs had positive effects on improving mood states, except a nonsignificant vigor–activity increase in the walk alone group. The three programs did not exhibit significant differences in changes of restorative benefits in physiological and psychological measures except for a significant difference in changes in sympathetic nervous activity between the walk alone group and guided group, which showed the guided program performed better in mitigating the sympathetic nervous system activity of participants.

## Figures and Tables

**Table 1:** Reviewed literature on types of programs in forest therapy studies.

Programs	References
Alone (participants walking and/or watching in a forest individually)	Beil and Hanes [32]; Komori et al. [33]; Lee et al. [16]; Lee et al. [18]; Mao et al. [5]; Park et al. [20]; Park et al. [27]; Park et al. [10]; Park et al. [22]; Song et al. [8]; Takayama et al. [21].
Guided (participants interacting with a guide)	Chen, Yu and Lee [34]; Ochiai et al. [7]; Ochiai et al. [11]; Song et al. [24]; Yu et al. [31].

**Table 2:** Timeframe and activities for the guided forest therapy program.

Locations	Activities	Senses	Time (min)
1	Warm-up and departing the aromatherapy room for Location 2		5
2	Starting point: explaining the sensory activities in the forest therapy program (visual, auditory, olfactory, and tactile)		5
3	Strolling in a bamboo forest	Visual and tactile	10
4	Admiring forests, listening to a river, and exploring sounds from insects and birds	Visual and auditory	10
5	Cooling down with stream water; breathing and stretching exercises	Tactile and olfactory	15
	Break		10
6	Walking on a Japanese cedar path and experiencing the forest’s density and serenity	Visual and auditory	10
7	Embracing trees and enjoying the forest air	Tactile and olfactory	10
8	Listening to nature in the quiet forest and sharing sounds with other participants	Auditory	10
9	Wrap-up		5

**Table 3:** Demographic characteristics of study participants (*N* = 99).

Demographics		*N* (%)
Gender	Female	40 (40.4)
	Male	59 (59.6)
Age	21–30	38 (38.4)
	31–50	14 (14.1)
	51–65	41 (41.4)
	≥66	6 (6.1)
Education	Graduate school	17 (17.2)
	College	58 (58.6)
	High school	17 (17.2)
	Middle school and below	7 (7.1)
Living area	Urban	45 (45.5)
	Suburban	42 (42.4)
	Rural	9 (9.1)
	Others	3 (3.0)
Last time to visit nature	Within 1 week	38 (38.4)
	Between 1 week and half month	30 (13.5)
	Between half month to 1 month	8 (8.1)
	Over 1 month	23 (23.3)

**Table 4:** Descriptive analysis among three groups.

	Indicators	*N*	Walk Alone Group (Control, C)		*N*	Self-Guided Group/Map (M)		*N*	Guided Group (G)	
			Pre	Post		Pre	Post		Pre	Post
Physiological	HR (bpm)	25 ^a^	71.7 ± 10.3	73.0 ± 11.5	33 ^b^	70.5 ± 8.1	74.1 ± 11.4	28	75.1 ± 10.8	76.8 ± 13.4
	SBP (mmHg)	25 ^a^	123.0 ± 14.7	118.2 ± 13.6	33 ^b^	119.4 ± 18.6	115.8 ±16.4	28	116.3 ± 14.1	110.1 ± 12.3
	DBP (mmHg)	25 ^a^	75.0 ± 7.9	72.7 ± 6.9	33 ^b^	74.5 ± 7.1	72.5 ± 7.5	28	71.7 ± 7.5	71.0 ± 7.8
	RFa (bpm^2^)	25 ^a^	1.4 ± 0.8	1.7 ± 0.8	33 ^b^	1.6 ± 1.0	1.4 ± 0.9	28	1.5 ± 0.6	1.4 ± 0.8
	LFa (bpm^2^)	25 ^a^	1.2 ± 0.7	1.4 ±0.6	33 ^b^	1.3 ±0.7	1.2 ± 0.7	28	1.4 ± 0.5	1.2 ± 0.4
	LFa/RFa	25 ^a^	1.0 ± 0.5	0.9 ± 0.4	33 ^b^	1.0 ± 0.5	1.0 ± 0.5	28	1.1 ± 0.5	1.0 ± 0.4
Psychological	T–A	37	4.2 ± 5.2	1.4 ± 2.7	34	6.5 ± 5.9	3.2 ± 3.5	28	6.9 ± 6.1	1.9 ± 2.9
	D–D	37	4.3 ± 6.9	1.3 ± 3.2	34	6.2 ± 6.4	2.1 ± 3.4	28	6.0 ± 6.4	1.9 ± 3.2
	A–H	37	3.6 ± 6.1	1.7 ± 2.8	34	4.7 ± 5.4	2.4 ± 2.5	28	4.3 ± 5.0	2.0 ± 1.9
	C–B	37	4.4 ± 4.0	1.8 ± 2.3	34	6.4 ± 4.5	2.6 ± 2.7	28	6.8 ± 4.6	2.3 ± 2.6
	F–I	37	5.9 ± 5.0	4.1 ± 4.3	34	6.6 ± 5.4	4.4 ± 3.0	28	7.5 ± 5.2	5.1 ± 3.9
	V–A	37	15.4 ± 4.8	15.6 ± 4.8	34	13.3 ± 5.0	15.1 ± 4.6	28	12.8 ± 4.5	15.1 ± 4.2

Abbreviations: HR, heart rate; SBP, systolic blood pressure; DBP, diastolic blood pressure; RFa, respiratory frequency area (parasympathetic nervous system activity measure); LFa, low frequency area (sympathetic nervous system activity measure); LFa/RFa (measure of sympathovagal balance); T–A, tension–anxiety; D–D, depression–dejection; A–H, anger–hostility; C–B, confusion–bewilderment; F–I, fatigue–inertia; V–A, vigor–activity. Note: (^a^) 12 participants did not have physiological measurements taken; (^b^) 1 participant did not have physiological measurements taken.

**Table 5:** Paired-sample *t*-test results of physiological responses in the three programs.

**Group C**	**Pre**	**Post**	***t***	***p***	**Change (%)**
HR (bpm)	71.7 ± 10.3	73.0 ± 11.5	0.872	0.392	1.73
SBP (mmHg)	122.8 ± 14.7	118.2 ± 13.6	−2.741	0.011 *	−3.78
DBP (mmHg)	75.0 ± 7.9	72.7 ± 6.9	−1.945	0.064	−3.09
RFa (bpm^2^)	1.4 ± 0.8	1.7 ± 0.8	2.021	0.055	21.13
LFa (bpm^2^)	1.2 ± 0.7	1.4 ±0.6	2.184	0.039 *	15.45
LFa/RFa	1.0 ± 0.5	0.9 ± 0.4	−0.793	0.436	−6.93
**Group M**	**Pre**	**Post**			
HR (bpm)	70.5 ± 8.1	74.1 ± 11.4	3.413	0.002 **	5.12
SBP (mmHg)	119.4 ± 18.6	115.8 ±16.4	−2.832	0.008 **	−3.04
DBP (mmHg)	74.5 ± 7.1	72.5 ± 7.5	−2.502	0.018*	−2.70
RFa (bpm^2^)	1.6 ± 1.0	1.4 ± 0.9	−1.332	0.192	−10.76
LFa (bpm^2^)	1.3 ±0.7	1.2 ± 0.7	−0.933	0.358	–6.87
LFa/RFa	1.0 ± 0.5	1.0 ± 0.5	0.373	0.711	4.12
**Group G**	**Pre**	**Post**			
HR (bpm)	75.1 ± 10.8	76.8 ± 13.4	1.334	0.193	2.24
SBP (mmHg)	116.3 ± 14.1	110.1 ± 12.3	−4.360	0.000 ***	−5.31
DBP (mmHg)	71.7 ± 7.5	71.0 ± 7.8	−0.0733	0.470	−0.93
RFa (bpm^2^)	1.5 ± 0.6	1.4 ± 0.8	−0.0556	0.583	−5.41
LFa (bpm^2^)	1.4 ± 0.5	1.2 ± 0.4	−1.850	0.075	−11.76
LFa/RFa	1.1 ± 0.5	1.0 ± 0.4	−1.039	0.308	−6.60

Abbreviations: HR, heart rate; SBP, systolic blood pressure; DBP, diastolic blood pressure; RFa, respiratory frequency area (parasympathetic nervous system activity measure); LFa, low frequency area (sympathetic nervous system activity measure); LFa/RFa (measure of sympathovagal balance). Note: *p < 0.05, **p < 0.01, ***p < 0.001.

**Table 6:** Comparisons of physiological response changes among the three programs.

Physiological Changes	Group	Mean ± SD	Change (%)	*F*	*p*	η_p_^2^	Post-hoc
△HR (bpm)	C	1.24 ± 7.11	1.73	1.527	0.223	0.035	
	M	4.12 ± 6.93	5.12				
	G	1.67 ± 6.66	2.24				
△SBP (mmHg)	C	−4.64 ± 8.47	−3.78	0.594	0.555	0.014	
	M	−3.974 ± 8.05	−3.04				
	G	−61.81 ± 7.50	−5.31				
△DBP (mmHg)	C	−2.321 ± 5.96	−3.09	0.852	0.430	0.020	
	M	−2.24 ± 5.15	−2.70				
	G	−0.68 ± 4.90	−0.93				
△RFa (bpm^2^)	C	0.29 ± 0.73	21.13	3.102	0.050	0.070	
	M	−0.16 ± 0.68	−10.76				
	G	−0.08 ± 0.73	−5.41				
△LFa (bpm^2^)	C	0.19 ± 0.44	15.45	3.552	0.033 *	0.079	C > G
	M	−0.10 ± 0.60	−6.87				
	G	−0.16 ± 0.46	−11.76				
△LFa/RFa	C	−0.07 ± 0.45	−6.93	0.527	0.592	0.013	
	M	0.04 ± 0.57	4.12				
	G	0.07 ± 0.47	−6.60				

Abbreviations: HR, heart rate; SBP, systolic blood pressure; DBP, diastolic blood pressure; RFa, respiratory frequency area (parasympathetic nervous system activity measure); LFa, low frequency area (sympathetic nervous system activity measure); LFa / RFa (measure of sympathovagal balance). Note: * *p* < 0.05.

**Table 7:** Paired-sample t-test results of psychological responses in the three programs.

**Group C**	**Pre**	**Post**	***t***	***p***	**Change (%)**
T–A	4.2 ± 5.2	1.38 ± 2.66	−3.796	0.001 **	−66.83
D–D	4.3 ± 6.9	1.27 ± 3.21	−3.277	0.002 **	−70.26
A–H	3.6 ± 6.1	1.68 ± 2.75	−2.054	0.047 *	−52.94
C–B	4.4 ± 4.0	1.78 ± 2.25	−4.401	0.000 ***	−59.64
F–I	5.9 ± 5.0	4.14 ± 4.32	−2.286	0.028 *	−29.71
V–A	15.4 ± 4.8	15.62 ± 4.76	0.310	0.758	1.76
Group M	Pre	Post			
T–A	6.5 ± 5.9	3.15 ± 3.46	−4.307	0.000 ***	−51.76
D–D	6.2 ± 6.4	2.06 ± 3.35	−5.409	0.000 ***	−66.99
A–H	4.7 ± 5.4	2.44 ± 2.45	−3.088	0.004 **	−45.41
C–B	6.4 ± 4.5	2.62 ± 2.70	−6.760	0.000 ***	−59.32
F–I	6.6 ± 5.4	4.44 ± 3.03	−2.421	0.021 *	−32.63
V–A	13.3 ± 5.0	15.09 ± 4.59	2.180	0.036 *	13.80
Group G	Pre	Post			
T–A	6.9 ± 6.1	1.9 ± 2.9	−5.549	0.000 ***	−71.99
D–D	6.0 ± 6.4	1.9 ± 3.2	−5.079	0.000 ***	−68.29
A–H	4.3 ± 5.0	2.0 ± 1.9	−2.982	0.006 **	−54.31
C–B	6.8 ± 4.6	2.3 ± 2.6	−6.576	0.000 ***	−65.63
F–I	7.5 ± 5.2	5.1 ± 3.9	−2.698	0.012 *	−31.87
V–A	12.8 ± 4.5	15.1 ± 4.2	2.444	0.021 *	18.10

Abbreviations: T–A, tension–anxiety; D–D, depression–dejection; A–H, anger–hostility; C–B, confusion–bewilderment; F–I, fatigue–inertia; V–A, vigor–activity. Note: * *p* < 0.05, ** *p* < 0.01, *** *p* < 0.001.

**Table 8:** Differences of mood state change scores among the three programs.

Physiological Changes	Group	Mean ± SD	Change (%)	F	*p*	η_p_^2^	Post Hoc
ΔT–A	C	−2.78 ± 4.46	−66.83	1.869	0.160	0.037	
	M	−3.38 ± 4.58	−51.76				
	G	−4.96 ± 4.73	−71.99				
ΔD–D	C	−3.00 ± 5.57	−70.26	0.628	0.536	0.013	
	M	−4.18 ± 4.50	−66.99				
	G	−4.07 ± 4.24	−68.29				
ΔA–H	C	−2.321 ± 5.96	−52.94	0.087	0.917	0.002	
	M	−2.24 ± 5.15	−45.41				
	G	−0.68 ± 4.90	−54.31				
ΔC–B	C	−2.62 ± 3.62	−59.64	2.290	0.107	0.046	
	M	−3.82 ± 3.30	−59.32				
	G	−4.43 ± 3.56	−65.63				
ΔF–I	C	−1.76 ± 4.67	−29.71	0.143	0.867	0.003	
	M	−2.15 ± 5.17	−32.63				
	G	−2.39 ± 4.69	−31.87				
ΔV–A	C	0.27 ± 5.31	1.76	1.491	0.230	0.030	
	M	1.82 ± 4.88	13.80				
	G	2.32 ± 5.03	18.10				

Abbreviations: T–A, tension–anxiety; D–D, depression–dejection; A–H, anger–hostility; C–B, confusion–bewilderment; F–I, fatigue–inertia; V–A, vigor–activity.

## Data Availability

The data are available upon request from the corresponding author.
